# “Modified” Liquid–Liquid Displacement Porometry and Its Applications in Pd-Based Composite Membranes

**DOI:** 10.3390/membranes8020029

**Published:** 2018-06-08

**Authors:** Lei Zheng, Hui Li, Haijun Yu, Guodong Kang, Tianying Xu, Jiafeng Yu, Xinzhong Li, Hengyong Xu

**Affiliations:** 1 Dalian National Laboratory for Clean Energy, Dalian Institute of Chemical Physics, Chinese Academy of Sciences, Dalian 116023, China; zlei@dicp.ac.cn (L.Z.); yuhj@dicp.ac.cn (H.Y.); kangguod@dicp.ac.cn (G.K.); xutianying@dicp.ac.cn (T.X.); yujf@dicp.ac.cn (J.Y.); 2 University of Chinese Academy of Sciences, Beijing 100049, China; 3 School of Materials Science and Engineering, Harbin Institute of Technology, Harbin 150001, China

**Keywords:** MLLDP, porous membrane, pore mouth size distribution, dense Pd membrane, defect distribution

## Abstract

For H_2_ separation by Pd-based composite membranes, the pore mouth size distribution of the porous support immediately affects the quality of the deposited layer, including continuity and defect/pinhole formation. However, there is a lack of convenient and effective methods for characterization of pore mouth size of porous supports as well as of defect distribution of dense Pd-based composite membranes. Here we introduce a novel method by modifying conventional liquid–liquid displacement porometry. When the pore tunnels are filled with Liquid B and the outer surface is occupied by Liquid A, the reopening of the pore mouth depends on the pressure of Liquid B and the interfacial tension at the position of the pore mouth, from which the pore mouth size can be determined according to the Young–Laplace equation. Our experimental tests using this method with model samples show promising results, which are well supported by those obtained using FESEM (fild emission scanning electron microscope), AFM (atomic force microscope), and conventional liquid–liquid displacement porometry. This novel method can provide useful information for not only surface coatings on porous substrates but also for modification of dense membrane defects; thus, broad utilizations of this technique can be expected in future study.

## 1. Introduction

Membrane technology has been extensively investigated in energy- and environment-related issues, such as H_2_ separation, natural gas purification, water treatment, etc. The functional layers of either inorganic or organic materials are often supported on a porous substrate such as α-alumina, zeolites, or stainless steel. Within this asymmetric structure, the porous substrate provides mechanical support and, thus, the thickness of functional layers can be significantly reduced. Conventional Pd metal tubes have been applied in the semiconductor and electronics industries, but are at least 100 micrometers. By forming a composite membrane on a porous alumina or stainless steel substrate, the thickness of the palladium layer can be reduced to several micrometers, which greatly lowers the cost and improves the hydrogen permeability [1,2]. For porous materials used as a membrane substrate, the size of the pore mouth is more of a concern than that of the pore throat [3], as it immediately determines the quality of the deposited layer including continuity and defect/pinhole formation [4,5,6,7,8,9]. For Pd composite membranes, major defects of the porous substrate lead to increased thickness as well as crack/pinhole formations [1,10]. On the other hand, the exfoliation of the Pd layer from the substrate can easily occur at a pore mouth size below ca. 20 nm due to a weak adhesion effect [11].

Currently, there exist several techniques for the determination of the pore size distribution of porous materials, as elaborated in Table 1, but there is still lack of efficient methods for pore mouth size characterization. Direct observation methods including SEM [12], FESEM [13], ESEM (enviromental scanning electron microscope) [14], and AFM [15] can provide general surface information of porous materials directly, but they are expensive and time consuming. Moreover, they provide only local information of a specific area (ca. 100 μm^2^) and require broken pieces of membranes.

Recently Krantz et al. [13] reported a detailed description of indirect methods, which can be separated into three groups, i.e., GAD (gas adsorption/desorption) [22], permporometry [17], and EP (evapoporometry) [13] based on the Kelvin equation; thermoporometry [19] based on the Gibbs–Thompson equation; and mercury intrusion porometry [16], BPM (bubble point method) [20], MBPM (“modified” bubble point method) [3], and LLDP (liquid–liquid displacement porometry) [21] based on the Young–Laplace equation. GAD, EP, and thermoporometry appear effective for average pore size measurement. GAD detects not only continuous pores but also dead-end pores. EP is a simple approach based on gravimetric measurement which does not require any assumed model for the pore geometry. Permporometry is based on capillary condensation of vapor and the blocking effect of permeation of a noncondensable gas, which measures pore throat size distribution. Nanopermorometry was also reported and is a method based on the Kelvin equation to characterize the Kelvin diameter of porous membranes [18], where the results denote a bimodal membrane structure described by a dense matrix and highly permeable regions.

Mercury intrusion porosimetry [16] provides pore throat size information, but it detects not only the continuous pores but also the dead-end pores. BPM [20] and LLDP [21], due to their convenience, have been widely applied in pore size measurements. These two methods work via a straightforward mechanism and the pressure required to reopen the pore depends on the capillary force in the pore. With the increase of pressure, the pores reopen from big to small ones gradually. Usually, the maximum capillary force throughout the pore is at the pore throat along the pore tunnel, and, thus, these two methods measure the pore throat size distribution. Huang et al. [3] reported MBPM to determine the pore mouth size distribution, a method which is modified from conventional BPM. Liquid is added to the pore mouth while the pore tunnel is purged with gas under pressure, and the closure of the pore depends on the capillary force at the pore mouth when gradually decreasing the pressure. However, this method requires relatively high pressures to measure small pore mouth sizes due to high gas–liquid surface tensions, e.g., 2.9 MPa for a pore size of 0.1 μm when using water as the impregnating agent. This increases the sealing difficulty and, in addition, the “entrainment phenomenon” (bubbling of liquid due to high-pressure gas) during experiments leads to large errors in pore size analysis, especially in case of high gas fluxes.

**Figure 1:**
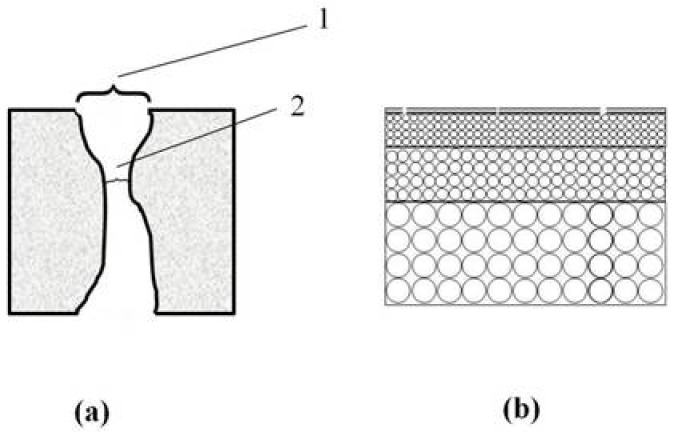
(**a**) Schematic of a pore tunnel. 1, pore mouth; 2, pore throat; (**b**) Schematic of dense membrane defects.

In this work, we introduce convenient “modified” liquid–liquid displacement porometry (MLLDP) to measure the pore mouth size. This method can operate under reasonably low pressures for a wide spectrum of pore sizes due to the relatively lower liquid–liquid interfacial tensions than gas–liquid surface tensions. In addition, the “entraining phenomenon” can be eliminated in the MLLDP method. This novel technique is especially suitable for pore mouth size analysis of multichannel membranes due to the recyclability of the testing liquid. The defect distribution of supported palladium membranes can also be characterized by this novel method, assuming straight defect pores in the thin dense layer (Figure 1).

**Figure 2:**
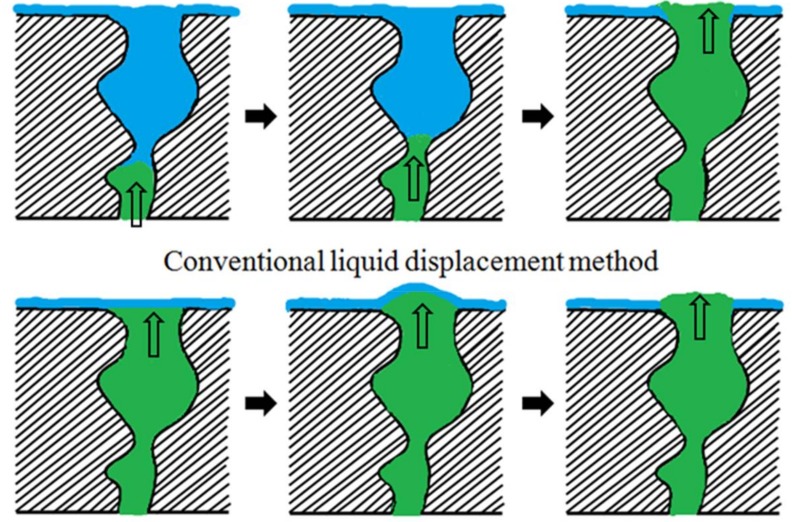
Schematic of the conventional and “modified” liquid–liquid displacement methods (Liquid A in blue, Liquid B in green).

Figure 2 shows a comparison between conventional and “modified” liquid–liquid displacement porometry. In conventional liquid–liquid displacement porometry, the pore tunnels of porous samples are first filled with Liquid A, and then purged with immiscible Liquid B. With the increase of pressure, Liquid B is gradually pushed outwards until the liquid–liquid interface reaches the pore throat. Once the pressure is high enough to overcome the interfacial tension of the liquid at the pore throat, the pore tunnel would be reopened.

In “modified” liquid–liquid displacement porometry, however, the pore tunnels are first filled with Liquid B and then the outer surface is occupied by Liquid A. Therefore, the liquid–liquid interface appears at the pore mouth instead of the pore throat. Once the pressure of Liquid B is high enough to overcome the interfacial tension at the pore mouth, the pore would be reopened. It should be noted that the measurement errors in conventional liquid–liquid displacement porometry (LLDP) due to resistance in the sublayer [21] do not apply to this “modified” liquid–liquid displacement porometry (MLLDP). According to the Young–Laplace equation, due to lower liquid–liquid interfacial tensions used in the MLLDP method, it is possible to measure relatively small pores with reasonably low pressure which does not damage the porous material. In addition, the low-pressure operation relaxes the requirements for equipment sealing.

## 2. Methods, Materials, and Apparatus

### 2.1. “Modified” Liquid–Liquid Displacement Porometry (MLLDP)

MLLDP is an indirect method based on the Young–Laplace equation, and the pore diameter can be calculated as [3]

(1) $$d=\frac{4\gamma \cos\theta }{P}$$

where γ is the interfacial tension coefficient between the liquid pair, θ is the contact angle of the penetrating agent on the pore wall, and *P* is the critical pressure to reopen the pore mouth. When the wetting effect is perfect, the contact angle can be assumed as 0. The pore mouths will be reopened from bigger to smaller ones with increasing pressure of the penetrating agent. A pressure–flux curve can be obtained by monitoring the pressure and flux of the penetrating agent through the pore tunnels. The theoretical derivation of the distribution curve from experimental pressure–flux data was introduced by Grabar et al. [23] and later applied by McGuire et al. [24] and Piątkiewicz et al. [25]. This is based on the assumption of cylindrical and separated pores with various diameters for the real pore structure of the porous membranes. In addition, a continuous distribution function *f(D)* is assumed for the varying sizes of the pores. 

Based on the Hagen–Poiseuille equation, the liquid flow through pore tunnels can be described as

(2) $$Q=\frac{n\pi r^{4}\Delta P}{8\mu l\tau }$$

Then, the pore mouth size distribution can be calculated using the following equation [3,16,20]:

(3) $$f\left(r\right)=\left(\frac{dQ}{d\left(\Delta P\right)}-\frac{Q}{\Delta P}\right)\frac{1}{r^{5}C_{2}}$$

where *Q* is the liquid flux, μ is the viscosity of the penetrating agent, *r* is the pore radius, *l* is the thickness of the porous material, τ is the pore curvature, *n* is the number of pores that can be opened, *∆P* is the operation pressure, and *C*_2_ is a constant number.

### 2.2. Materials

A wide range of pore sizes can be determined with different immiscible liquid systems depending on the interfacial tensions. Table 2 shows that the operation pressures required for MBPM are one or two orders of magnitude higher than that required for MLLDP, due to higher gas–liquid surface tensions than liquid–liquid interfacial tensions.

Well-defined membranes with a regular noninterconnected pore structure and narrow pore size distribution can be considered as standard to check the feasibility of pore size characterization methods. In this study, 100 nm Anopore^TM^ (Whatman, Maidstone, UK) membranes were adopted for validating the MLLDP method. These membranes comprise a thin layer of symmetric structure without any support layer. Therefore, the pressure difference was kept below 1 bar during MLLDP analysis to maintain integrity. MLLDP was also extended to characterization of commercially available symmetric PVDF membranes (polyvinylidene fluoride) fabricated by the non-solvent-induced phase separation method, single tubular and multichannel tubular ceramic membranes (commercial), as well as the defect size of supported Pd membranes prepared in-house by the electroless-plating method [26]. The geometrical details of these materials and operational characteristics during MLLDP analysis are depicted in Table 3.

### 2.3. Apparatus

**Figure 3:**
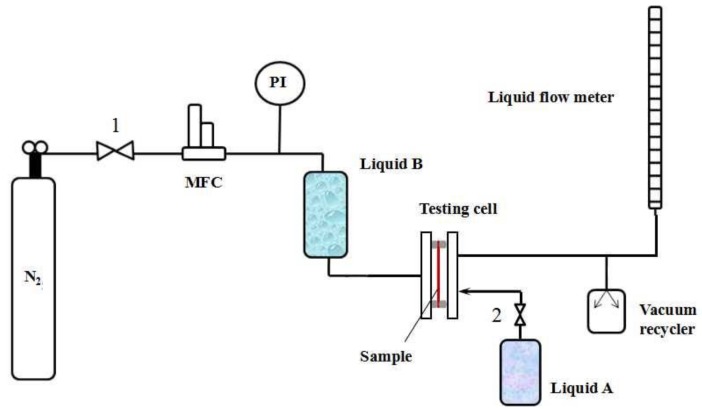
Schematic of apparatus for MLLDP measurements of an Anopore^TM^ membrane. MFC, mass flow controller. PI, pressure indicator.

**Figure 4:**
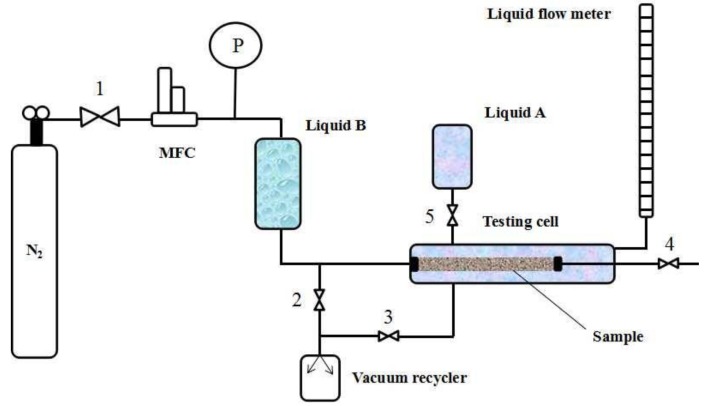
Schematic of apparatus for MLLDP measurements of a single-channel membrane. MFC, mass flow controller. PI, pressure indicator.

**Figure 5:**
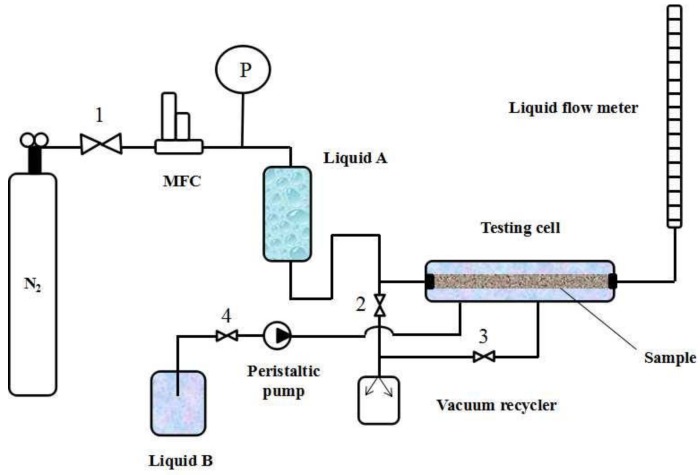
Schematic of apparatus for MLLDP measurements of a multichannel membrane. MFC, mass flow controller. PI, pressure indicator.

Figure 3 shows the MLLDP apparatus designed for pore mouth size characterization of Anopore^TM^ membranes while Figure 4 shows the apparatus for characterization of PVDF membranes, single tubular ceramic membranes, and dense Pd membranes (defects). The MLLDP apparatus shown in Figure 3 and Figure 4 have different testing cells which can be used to fulfill the aim of characterizing samples with different shapes. For these membranes, a functional layer is deposited on the outer surface of porous substrates. For multichannel tubular ceramic membranes, the functional layer is deposited on the inner surface of tubes, and the pore mouth size distribution of the inner surface is what we are concerned with. Therefore, a distinct testing cell was designed for the pore mouth size distribution of multichannel membranes, as shown in Figure 5. Note that the apparatus in Figure 4 is also applicable for conventional BPM measurements and conventional LLDP measurements for pore throat characterization due to the similarity in the operations.

Take the testing of a single-channel ceramic membrane as an example (test rig in Figure 4); the operating procedure is as follows:

1. The dry sample is mounted in the testing cell (drying procedure and pretreatment are important for porous materials [18,27,28]; thus, samples were carefully dried without changing the pore structure);
2. Liquid B (acting as penetrating agent) from the reservoir fills the lumen of the sample by opening Valve 4 for a short time;
3. The pressure of Liquid B is increased to 3 bar by increasing the N_2_ gas pressure while applying a negative pressure of −0.8 bar in the shell side by using a vacuum recycler (opening Valve 3) in order to achieve complete wetting of pore tunnels with Liquid B; this step lasts for at least 30 min;
4. Liquid A (acting as impregnating agent) from the reservoir is poured into the shell side by opening Valve 5 until the remaining Liquid B on the outer surface of the sample is completely replaced with Liquid A, i.e., until there is no Liquid B observed in the outflow (Liquids A and B can be easily distinguished by color), then Valve 5 is closed; Note that Liquid A is kept under ambient pressure and at a low flow rate below ca. 10 mL/min in order to avoid penetration of Liquid A into membrane pores;
5. The pressure of Liquid B increases step by step and a pressure–flux curve can be obtained by monitoring the N_2_ gas pressure at a step of 0.05–0.1 bar and flux of Liquid B through the pore tunnels (measured by bubble flow meter). The flow rate is recorded until a steady-state flux is achieved. The testing cell is kept at RT during the operation.

For LLDP measurement, the operation procedure is as below:

1. The dry sample is mounted in the testing cell;
2. Liquid A (acting as impregnating agent) from the reservoir is poured into the shell side by opening Valve 5 for a short time;
3. The sample is impregnated in Liquid A for a period of 30 min to 1 h in order to achieve complete wetting of pore tunnels with Liquid A;
4. Liquid B (acting as penetrating agent) from the reservoir fills the lumen of the sample by opening Valve 4 for a short time;
5. The pressure of Liquid B increases step by step and a pressure–flux curve can be obtained by monitoring the N_2_ gas pressure at a step of 0.05–0.1 bar and flux of Liquid B through the pore tunnels (measured by bubble flow meter). The flow rate is recorded until a steady-state flux is achieved. The testing cell is kept at RT during the operation.

It can be seen that Liquids A and B act as impregnating and penetrating agent, respectively, in cases of both MLLDP and LLDP measurement. In the former, Liquid B first fills the pore tunnels of the sample and then Liquid A occupies the outer surface of sample such that Liquids A and B contact each other at the position of the pore mouth. Liquid B penetrates through the pore tunnels when the pressure is high enough to overcome the interfacial tension at the pore mouth according to the Young–Laplace equation. In the latter, Liquid A first fills the pore tunnels of the sample and then Liquid B penetrates through the pore tunnels when the pressure is high enough to overcome the interfacial tension at the position of the pore throat according to the Young–Laplace equation. 

### 2.4. Error Analysis

The error in the pore diameter for techniques based on the Young–Laplace Equation (1) is strongly dependent on the accuracy in determining pressure values. This also applies to BPM, MBPM, LLDP, and MLLDP methods.

According to the Young–Laplace Equation (1), the fractional error in the diameter *(∆d/d)* that results from the fractional error in the pressure analysis is given by

(4) $$\frac{\Delta d}{d}=\frac{\Delta P}{P}$$

**Figure 6:**
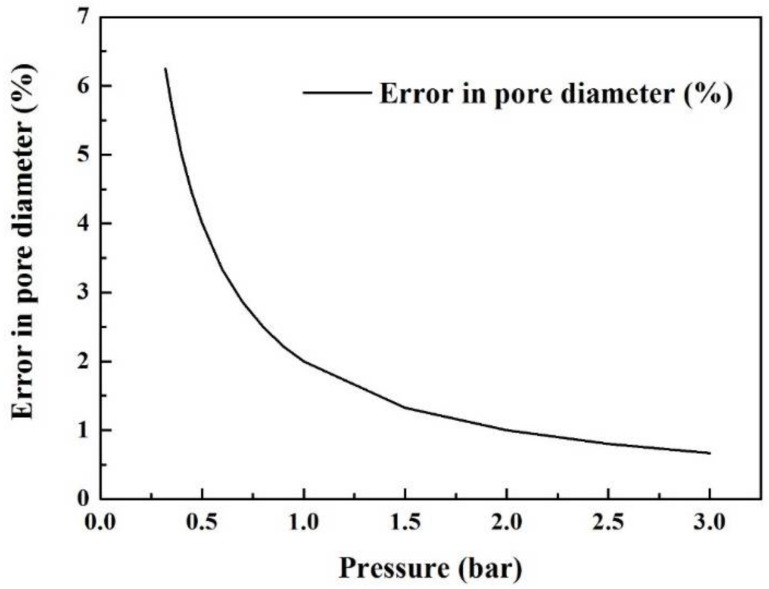
Percent error in the pore diameter as a function of the operation pressure.

In should be emphasized that the pressure analysis error due to the difference in liquid levels between the reservoir of Liquid B and flow meter (Figure 4) has to be taken into account. The liquid level in the bubble flow meter may fluctuate frequently with the flow in and out during the measurement, and needs to be kept at a similar level to the reservoir of Liquid B in order to minimize corresponding pressure differentials. In this study, such a level difference is deliberately kept within ±20 cm during the operation process, which corresponds to a low pressure differential of ca. 0.02 bar. The pressure of Liquid B is then set above 0.32 bar in order to ensure a fraction error in the pressure analysis *(∆P/P)* of less than ca. 6%. The fractional error in the diameter decreases with the increase of operational pressures, as shown in Figure 6.

As with other techniques, the accuracy of MLLDP decreases with increasing pore sizes. The analysis of large pores above ca. 100 µm requires measurements in a fairly low pressure range and thus degrades the accuracy.

## 3. Results and Discussions

### 3.1. Standard Membranes

#### 3.1.1. Anopore^TM^ Membranes (100 nm)

In order to verify the feasibility of this novel MLLDP method in measuring the pore mouth size distribution of porous membranes, 100 nm AnoporeTM membranes and 80 nm PVDF membranes were used as standard membranes and characterized by MLLDP. 

**Figure 7:**
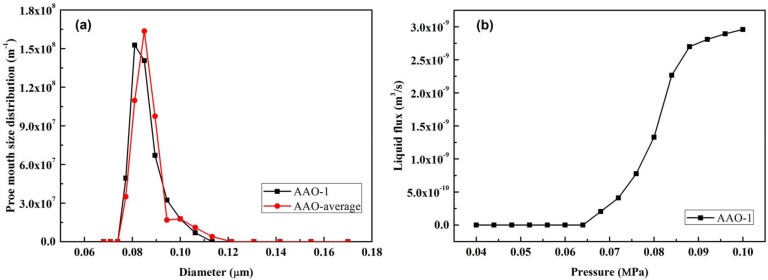
(**a**) Averaged pore mouth size distribution for Anopore^TM^ membranes derived from four replicate samples; (**b**) liquid flux and pressure relationship of AAO-1 membrane.

Figure 7a depicts the MLLDP analysis results for four replicate samples of the 100 nm Anopore^TM^ membrane, which indicates a good overlapping of the measurements with a fairly sharp pore mouth size distribution. This agrees well with the narrow pore size distribution of Anopore^TM^ membranes which exhibit regular columnar pore geometry. It should be mentioned that for the columnar pore geometry of Anopore^TM^ membranes, the pore mouth size is equal to the pore throat size. MLLDP analysis provides a number-averaged pore mouth size of 84.09 nm with σ = ±3.52 nm as listed in Table 4. The σ indicates the breadth of the pore mouth size distribution. The comparison in Table 5 shows a good agreement between MLLDP and other techniques, including evapoporometry [13], FESEM [13], SEM [29], AFM [30], and LLDP [21]. For example, FESEM [13] and SEM [21] analyses provide number-averaged pore mouth sizes of 87 nm and 83 nm, respectively, which are fairly close to that determined by MLLDP (84.09 nm). This has a strong relationship with the fact that MLLDP also provides a number-averaged pore mouth size. The pore diameter error with MLLDP analysis is kept within ca. 4%, which can be mainly ascribed to the proper selection of liquid pairs and minimization of errors in the pressure analysis. The slightly larger number-averaged pore size of 108 nm provided by AFM analysis [23] was attributed to a low lateral resolution resulting from the use of a 10 nm tip on AFM [4]. The mean-flow average pore size of 119 nm derived from LLDP measurement [21] is relatively larger than the number- or mass-averaged pore sizes determined by other techniques, which can be ascribed to the fact that the volume flow for cylindrical pores is proportional to *d*^4^ instead of *d*^2^ for pore mass.

#### 3.1.2. PVDF Membranes (80 nm)

**Figure 8:**
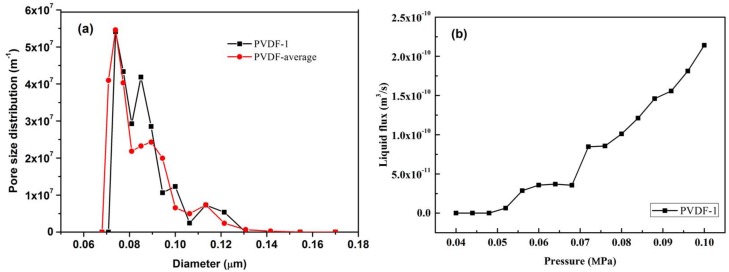
(**a**) Averaged pore mouth size distribution for PVDF membranes derived from six replicate samples; (**b**) liquid flux and pressure relationship of PVDF-1 membrane.

A representative run was carried out with six different sections of the same membrane tube and the results are reported in Figure 8 and Table 6. The graph shows a good overlapping of these replicate measurements, obtaining an average pore mouth size of 75.6 nm with σ = ±3.18 nm. This is in fair agreement with the nominal pore size of 80 nm provided by the supplier. The representative FESEM image in Figure 3 corroborated MLLDP analysis results although some large pores approaching 100 nm can also be identified on the membrane surface. It has been indicated previously that the high operation pressures required in LLDP measurements may lead to considerable compression of polymeric membranes and, thus, the shifting of the pore size distribution towards smaller pore diameters [4]. In this study, the liquid system was selected with discretion, i.e., isobutyl alcohol/water with a low interfacial tension of 1.7 mN/m, which helps maintain the pressure differential below 1 bar and thus minimize the compaction of PVDF membranes. This approach can be universal for MLLDP analysis of polymeric membranes.

The pore mouth size information of those standard membranes obtained by the MLLDP method are well in agreement with data from the supplier and literature reports. Therefore, MLLDP as a novel characterization method is able to provide the pore mouth size information of porous membranes.

### 3.2. Tubular Ceramic Substrates for Pd Composite Membranes

#### 3.2.1. Single Tubular ZrO_2_/γ-Al_2_O_3_/α-Al_2_O_3_ Membrane (30 nm)

**Figure 9:**
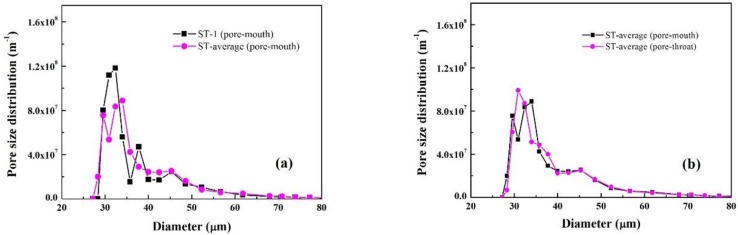
Characterization of single tubular ZrO_2_/γ-Al_2_O_3_/α-Al_2_O_3_ membranes with 3 replicate samples: (**a**) Averaged pore mouth size distribution in comparison with that for Sample 1; (**b**) Comparison of averaged pore mouth size distribution and averaged pore throat size distribution.

The pore mouth size distribution of commercially fabricated single tubular ZrO_2_/γ-Al_2_O_3_/α-Al_2_O_3_ membranes was characterized by MLLDP, as reported in Figure 9 and Table 7. Figure 9a shows a good overlapping of the three replicate measurements in a representative run. The average pore mouth size was determined as 31.98 nm with σ = ±1.06 nm, which is slightly larger than the nominal pore throat size of 30.05 nm with σ = ±7.02 nm measured by conventional LLDP, as shown in Figure 9b. This agrees well with the fact that pore mouth size is slightly larger than pore throat size. It should be noted that, due to resistance in the sublayer [21], the measurement error of conventional liquid–liquid displacement porometry (LLDP) was σ = ±7.02 nm. However, “modified” liquid–liquid displacement porometry (MLLDP) gave σ = ±1.06 nm, indicating that the measurement errors due to resistance in the sublayer do not apply to MLLDP, which is an improvement over LLDP.

**Figure 10:**
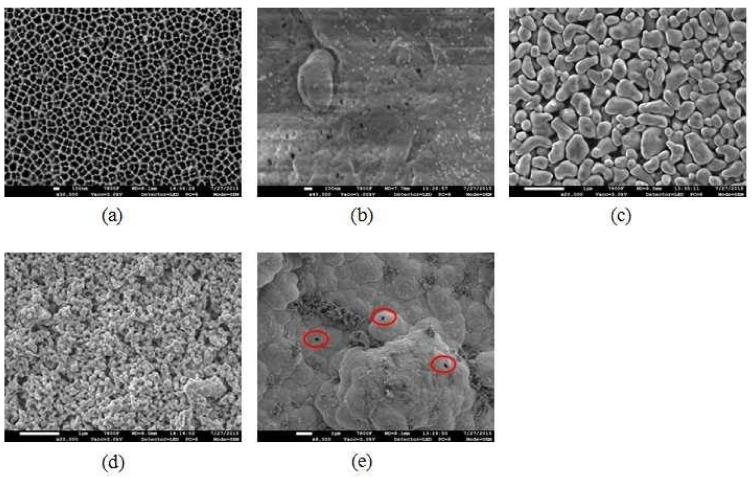
FESEM images: (**a**) 100 nm Anopore^TM^ membrane; (**b**) 80 nm PVDF membrane; (**c**) 30 nm single tubular ZrO_2_/γ-Al_2_O_3_/α-Al_2_O_3_ membrane (outer surface); (**d**) 100 nm multichannel tubular ZrO_2_/γ-Al_2_O_3_/α-Al_2_O_3_ membrane (inner surface); (**e**) defects of dense Pd composite membrane.

Inspection of the FESEM image in Figure 10c indicates a rather smooth surface with interconnected irregular-shaped pores. A wide range of pore sizes, particularly some large pores approaching up to 60 nm, can be discriminated on the membrane surface, which coincides well with the broad pore mouth size distribution determined by MLLDP as shown in Figure 9a. Note that the operation pressures applied during MLLDP or LLDP measurements will not affect the pore size distribution of ceramic membranes which exhibit strong mechanical strength. 

#### 3.2.2. Multichannel Tubular ZrO_2_/γ-Al_2_O_3_/α-Al_2_O_3_ Membrane (100 nm)

**Figure 11:**
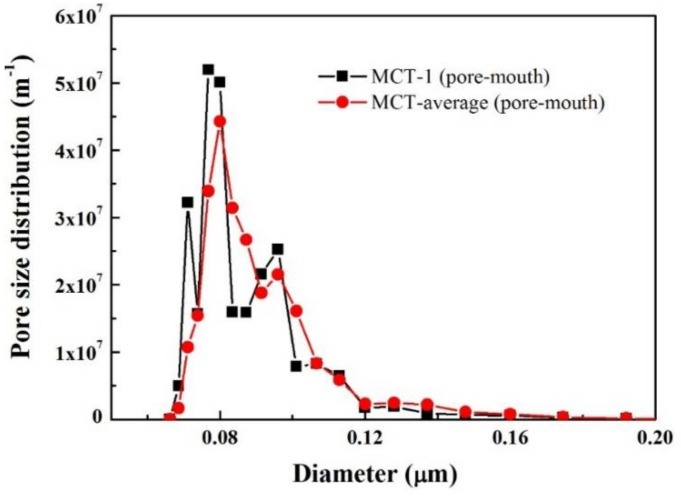
Averaged pore mouth size distribution for multichannel tubular ZrO_2_/γ-Al_2_O_3_/α-Al_2_O_3_ membranes derived from six replicate samples.

The pore mouth size distribution of multichannel tubular ZrO_2_/γ-Al_2_O_3_/α-Al_2_O_3_ membranes (commercial) was also examined by MLLDP. Figure 11 shows that three repeated measurements of the same sample exhibit excellent reproducibility with similar patterns of distribution and an obtained average pore mouth size of 80.09 nm (σ = ±2.72 nm), the results were summarized in Table 8. This is in good agreement with the nominal pore size of 100 nm. The representative FESEM image in Figure 10d indicates a relatively narrow distribution of pore sizes on the membrane surface, which corresponds well with the MLLDP results in Figure 11. Despite the rough inner surface of the multichannel tubular membranes as shown in Figure 10d, the pores are assumed to be perpendicular to the local surface, which is apparently the case in a short range of ca. 80 nm. This assumption seemingly did not affect the pore mouth size analysis by MLLDP, which shows good agreement with nominal values. It should be emphasized that MLLDP exhibits a great advantage in terms of recyclability of the testing liquid, in comparison with MBPM measurement where gas such as air or N_2_ acts as penetrant and, thus, cannot be easily recycled.

### 3.3. Defects of a Dense Pd Composite Membrane

**Figure 12:**
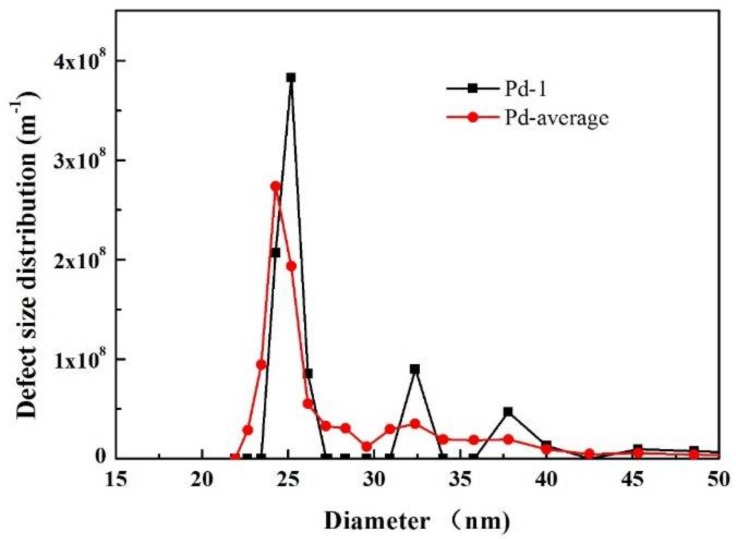
Averaged defect size distribution for Pd composite membranes derived from six replicate runs and four replicate samples.

Currently, there is lack of methods for defect size characterization of dense Pd composite membranes. Using the MLLDP analysis presented in this study, the defect size distribution was successfully obtained, as shown in Figure 12 and Table 9. The six repeated measurements of the same Pd composite membrane (prepared in-house by the electroless-plating method [26]) indicate excellent reproducibility with an average defect size around 24.44 nm (σ of ±0.25 nm). The low σ suggests a relatively narrow distribution of defect sizes. This is in excellent agreement with the analysis of representative FESEM images in Figure 10e with defect sizes in a narrow range of 20–40 nm. Figure 10e also shows that the defects exhibit irregular-shaped geometries on rough surfaces. It can be claimed that the surface roughness has an inconsiderable effect on defect size analysis. In order to examine the defect size in a low range of ca. 20–40 nm, the liquid system of isobutyl alcohol/water with low interfacial tension was selected with discretion, which renders the operation in a proper pressure range (0.32–3.0 bar) and thus helps improve the accuracy of MLLDP measurements. 

### 3.4. Advantages and Limitations of MLLDP

#### 3.4.1. Advantages of MLLDP

MLLDP exhibits great advantages over direct observation methods including SEM, FESEM, and AFM, since it can provide a general picture of the whole membrane surface instead of local information on a small area (ca. 100 µm^2^) which might not be representative. In addition, SEM, FESEM, and AFM require expensive dedicated equipment and broken pieces of samples for measurement. In comparison, MLLDP can be carried out with a simple setup at a much lower cost. The main instruments included are a mass flow controller (MFC) and a pressure gauge. In addition, the membrane sample can remain intact during the MLLDP measurement. Another important advantage of MLLDP over direct methods is that it can be used for characterization of irregular pore geometry instead of only well-defined pore structures.

Most indirect methods provide either the pore throat size distribution or average pore size, including LLDP, mercury porosimetry, BPM, GAD, permporometry, and EP. The recently developed MBPM can be used for the characterization of the pore mouth size distribution; however, it requires high operation pressures due to high surface tensions between gas and liquid. MLLDP presented in this study renders the possibility of characterizing a wide range of pore sizes at a much lower pressure as the interfacial tensions between liquid pairs is several orders of magnitude lower than surface tensions between gas and liquid. Thus, by selecting a liquid system with discretion, it is possible to reduce the operational pressure for thin and flexible membranes and determine the pore mouth size distribution at high accuracy, e.g., for polymeric membranes. Moreover, MLLDP can be extended to pore mouth size characterization of nanofiltration (NF) membranes and defect size analysis of dense membranes down to the nanometer scale. MLLDP analysis for defect characterization of Pd composite membranes can be regarded as a breakthrough technology in the relevant field.

Two other advantages of MLLDP are that it can be applied for characterization of both symmetric and asymmetric membranes, and it detects only continuous pores but not dead-end pores, thus providing more relevant information for flow analysis. 

#### 3.4.2. Limitations of MLLDP

As with other techniques, the accuracy of MLLDP decreases with increasing pore size. This has been suggested to be a common issue for other techniques including GAD and permporometry based on the Kelvin equation as well as LLDP based on the Young–Laplace equation. In MLLDP, the analysis of large pores above ca. 100 µm requires measurements in a fairly low pressure range and thus degrades the accuracy.

This technique employs a low pressure range; thus, small variations in the pressure can give higher relative errors in the measurement. The error in the pore diameter is highly sensitive to pressure analysis errors within Equation (1), which places urgency upon the accuracy of the pressure measurement. High-precision pressure analysis is thus a prerequisite for MLLDP measurement. In addition, the difference in liquid levels between the reservoir and liquid flow meter has to be minimized which may lead to some errors in the pressure analysis. Another limitation of MLLDP is that it assumes a noninterconnected, separated columnar pore structure, which may cause data interpretation problems for interconnecting pores on the surface. However, this is common for all other techniques mentioned above.

## 4. Conclusions

In the preparation of dense Pd composite membranes, the pore size distribution of the porous support immediately affects the quality of the membrane layer, including continuity and defect/pinhole formation. This work presents a novel method (MLLDP) to determine the pore mouth size distribution of porous supports and defect size distribution of Pd-based composite membranes by modifying the conventional liquid–liquid displacement method. MLLDP can be used to determine a wide spectrum of pore mouth sizes (defect sizes) under reasonably low pressures and thus achieve great accuracy and ease of operation, offering unique superiority compared with currently available technologies like FESEM, AFM, and the “modified” bubble-point method. Particularly, MLLDP exhibits great advantages for the characterization of multichannel tubular ceramic membranes in terms of recyclability of the testing liquid, and MLLDP analysis for defect characterization of dense membranes can be regarded as a breakthrough technology in the relevant field. It can thus be expected that this novel method will find wide applications in the surface analysis of porous materials as well as in defect analysis of dense membranes. 

## Figures and Tables

**Table 1:** Comparison of existing pore size characterization methods.

Methods		Equation	Pore Size Information	Ref.
Direct	SEM	-	Pore mouth	[12]
	AFM	-	Pore mouth	[15]
	ESEM	-	Pore mouth	[14]
	FESEM	-	Pore mouth	[13]
Indirect	GAD	Kelvin	Average	[16]
	Permporometry	Kelvin	Pore throat	[17]
	Nanopermporometry	Kelvin	Kelvin diameter	[18]
	EP	Kelvin	Average	[13]
	Thermoporometry	Gibbs–Thompson	Average	[19]
	BPM	Young–Laplace	Pore throat	[20]
	MBPM	Young–Laplace	Pore mouth	[3]
	LLDP	Young–Laplace	Pore throat	[21]
	Mercury intrusion porometry	Young–Laplace	Pore throat	[16]
	MLLDP	Young–Laplace	Pore mouth	This work

**Table 2:** A comparison of operation pressures required for MBPM and MLLDP (20 °C, cos θ = 1).

Method	System	Interfacial Tension (mN/m)	Pressure (MPa) Corresponding to Different Pore Diameters (μm)			
			0.2	0.1	0.05	0.025
MBPM	water–nitrogen	72.8	1.45	2.91	5.82	11.64
	ethanol–nitrogen	22.39	0.44	0.89	1.79	3.58
MLLDP	n-amyl alcohol ^a^/water	4.8	0.096	0.19	0.38	0.76
	isobutyl alcohol ^a^/water	1.7	0.034	0.068	0.13	0.27
	oil–aqueous phase ^b^/water	0.35	0.007	0.014	0.028	0.056

^a^ n-amyl alcohol and isobutyl alcohol are saturated with water at a volumetric proportion of 1:1; ^b^ oil–aqueous phase is prepared using isobutyl alcohol, methanol, and water at a volumetric proportion of 15:7:25.

**Table 3:** Geometrical details of materials investigated and operational characteristics during MLLDP analysis.

Materials	Shape	Geometry	Nominal Pore Size	Liquid System (B/A)	Δ*p* (bar)	Test Rig
Anopore^TM^ membrane	Planar	O.d.: 7 mm Thickness: 60 μm	100 nm	Isobutyl alcohol/water	0.4–1.0	Figure 3
PVDF membrane	Tubular	O.d.: 1.2 mm I.d.: 0.7 mmL: 40 cm	80 nm	Isobutyl alcohol/water	0.4–1.0	Figure 4
ZrO_2_/γ-Al_2_O_3_/α-Al_2_O_3_ membrane	Single tubular	O.d.: 13 mm I.d.: 11 mmL: 5 cm	30 nm	Isobutyl alcohol/water	0.6–3.0	Figure 4
ZrO_2_/γ-Al_2_O_3_/α-Al_2_O_3_ membrane	Multi-channel tubular	O.d.: 32 mm Channel i.d.: 4 mmL: 100 cmChannel no.: 19	100 nm	n-amyl alcohol/water	0.4–2.8	Figure 5
Defects of Pd/ZrO_2_/Al_2_O_3_ membrane	Tubular	O.d.: 13 mm I.d.: 11 mmL: 5 cm	-	Isobutyl alcohol/water	0.32–3.0	Figure 4

**Table 4:** Summary of MLLDP results for four replicate samples of nominal 100 nm Anopore^TM^ membrane.

Types	Membranes				Average	σ	Avg ± σ
	AAO-1	AAO-2	AAO-3	AAO-4			
Number-averaged pore mouth (nm)	80.95	80.95	89.47	85.00	84.09	3.52	84.09 ± 3.52

**Table 5:** Comparison of PSD (pore size distribution) measurements of nominal 100 nm Anopore^TM^ membrane with different methods.

Methods	Average	Pore Mouth/Pore Throat/Mean Pore Size	Averaged Pore Size (nm)	Ref.
EP *	Mass-average	Mean pore size	94 ± 14	[13]
	Number-average	Mean pore size	69 ± 34	[13]
FESEM	Number-average	Pore mouth	87 ± 10	[13]
SEM	Number-average	Pore mouth	83 ± 26	[29]
LLDP	Mean-flow average	Pore throat	119	[29]
AFM	Number-average	Pore mouth	108 ± 26	[30]
MLLDP	Number-average	Pore mouth	84.09 ± 3.52	This work

* EP: Evapoporometry.

**Table 6:** Summary of MLLDP results for six replicate samples of nominal 80 nm PVDF membrane.

Types	Tubes						Average	σ	Avg ± σ
	P-1	P-2	P-3	P-4	P-5	P-6			
Number-averaged pore mouth size (nm)	73.9	70.8	73.9	80.9	77.0	77.0	75.6	3.18	75.6 ± 3.18

**Table 7:** Summary of MLLDP results for three replicate samples of nominal 30 nm single tubular ZrO_2_/γ-Al_2_O_3_/α-Al_2_O_3_ membrane.

Types	Repeat Times			Average	σ	avg ± σ
	ST-1	ST-2	ST-3			
Number-averaged pore mouth (nm)	32.38	34	29.56	31.98	1.06	31.98 ± 1.06
Number-averaged pore throat (nm)	30.91	30.91	28.33	30.05	7.02	30.05 ± 7.02

**Table 8:** Summary of MLLDP results for three replicate samples of nominal 100 nm multichannel tubular ZrO_2_/γ-Al_2_O_3_/α-Al_2_O_3_ membrane.

Types	Repeat Times			Average	σ	avg ± σ
	MCT-1	MCT-2	MCT-3			
Number-averaged pore mouth (nm)	76.80	83.48	80.00	80.09	2.72	80.09 ± 2.72

**Table 9:** Summary of MLLDP results for six replicate runs of Pd composite membrane.

Types	Repeat Times						Average	σ	avg ± σ
	Pd-1	Pd-2	Pd-3	Pd-4	Pd-5	Pd-6			
Number-averaged defect size (nm)	24.29	24.29	25.11	24.29	24.29	24.29	24.44	0.25	24.44 ± 0.25
